# The Quality of Internet Websites for People Experiencing Psychosis: Pilot Expert Assessment

**DOI:** 10.2196/28135

**Published:** 2022-04-15

**Authors:** Kay Wilhelm, Tonelle Handley, Catherine McHugh, David Lowenstein, Kristy Arrold

**Affiliations:** 1 Discipline of Psychiatry, School of Medicine University of Notre Dame Sydney Australia; 2 Faculty of Medicine University of New South Wales Sydney Australia; 3 School of Medicine and Public Health University of Newcastle Callaghan Newcastle Australia; 4 Brain and Mind Centre University of Sydney Sydney Australia; 5 Tasmanian Health Service Hobart Australia

**Keywords:** psychosis, schizophrenia, DISCERN, quality, websites, mental health, Australia, health information, patients, consumers, accessibility, patient empowerment, reliability, eHealth, electronic health, website

## Abstract

**Background:**

Clinicians need to be able to assess the quality of the available information to aid clinical decision-making. The internet has become an important source of health information for consumers and their families.

**Objective:**

This study aimed to rate the quality of websites with psychosis-related information (to provide clinicians with a basis for recommending material to guide clinical decision-making with consumers and their families), using a validated instrument as well as a purpose-developed checklist, and consider improvement in quality over a 4-year period.

**Methods:**

Two measures of website quality were used: the DISCERN scale and the Psychosis Website Quality Checklist (PWQC). Terms related to psychosis, including “psychotic,” “psychosis,” “schizophrenia,” “delusion,” and “hallucination,” were entered into Google, and the first 25 results were analyzed. In total, 6 raters with varying health professional backgrounds were used to evaluate the websites across two time points: January-March 2014 and January-March 2018.

**Results:**

Of the 25 websites rated, only the 6 highest ranked websites achieved a DISCERN score, indicating that they were of “good” quality (51-62 out of a possible 75), while the mean score of the websites (mean 43.96, SD 12.08) indicated an overall “fair” quality. The PWQC revealed that websites scored highly on “availability and usability” (mean 16.82, SD 3.96) but poorly on “credibility” (mean 20.99, SD 6.68), “currency” (mean 5.16, SD 2.62), and “breadth and accuracy” (mean 77.87, SD 23.20). Most sites lacked information about early intervention, recreational drug use and suicide risk, with little change in content over time. Stating an editorial or review process on the website (found in 56% of websites) was significantly associated with a higher quality score on both scales (the DISCERN scale, *P*=.002; the PWQC, *P*=.006).

**Conclusions:**

The information on the internet available for clinicians to recommend to people affected by psychosis tended to be of “fair” quality. While higher-quality websites exist, it is generally not easy way to assess this on face value. Evidence of an editorial or review process was one indicator of website quality. While sites generally provided basic clinical information, most lacked material addressing weighing up risks and benefits of medication and alternatives, the role of coercive treatment and other more contentious issues. Insufficient emphasis is placed on detailed information on early intervention and importance of lifestyle modifications or how families and friends can contribute. These are likely to be the very answers that consumers and carers are seeking and this gap contributes to unmet needs among this group. We suggest that clinicians should be aware of what is available and where there are gaps.

## Introduction

Accessing information related to one’s illness and treatment enables consumers to discuss their health and treatments more confidently with their doctors [1], is central to the patient-centered health care delivery [2] and improves clinical outcomes [3]. It also offers an opportunity to seek information privately and at one’s own pace. This is particularly important in relation to psychotic disorders given the bewildering and anxiety-provoking nature of the symptoms themselves for the consumer, and their families or carers. Additionally, the impact of stigma, misinformation, and low levels of mental health literacy can impact have on outcomes of care [4,5].

Several mental illnesses, including schizophrenia, schizoaffective disorder, bipolar disorder, and some physical illnesses, can present with psychosis [6]. This is characterized by disrupted cognitive processes, delusions, hallucinations, and changes to speech and behavior patterns, which are bewildering for those who experience psychosis and their families. People experiencing psychosis, especially those with schizophrenia, often have pronounced fear and anxiety related to social engagement, and the internet offers a safe space to obtain mental health information without having to interact with others [7] and averts any possibility of feeling devalued.

The last decade has seen the internet become increasingly central to the dissemination of health care information. This technological transition has accelerated with the COVID-19 pandemic [8], where “accessing basic requirements like health and education” is increasingly achieved through web-based portals. While there is no conclusive evidence that the COVID-19 pandemic has increased the prevalence of severe psychiatric disorders such as psychosis, the associated distress, enforced social isolation, and related uncertainties may exacerbate symptoms [9]. Additionally, the pandemic has led to a decrease in face-to-face appointments with mental health professionals for those experiencing mental illness, leading to increased reliance on technology; for example, using the internet for both therapy sessions and informal information and support [9]. In one study, approximately 30% of patients with a psychotic disorder reported using the internet “a lot” during the pandemic; this rate was comparable to that of internet use by individuals with other severe mental illnesses [10].

The increasing reliance on the internet as a source of mental health information highlights the importance of clinicians being aware of what information is available on the internet. Even prior to the pandemic, approximately half of Australia’s 15 million internet users reported using internet to search for health-related information [11-13]. In mental health services, over 50% of people with a previous diagnosis of mental illness and access to the internet have used the internet to find diagnosis-related information [14,15]. Research has also shown that patients with schizophrenia differ from those with other mental illnesses in their internet search behavior, including the times of the day they use the internet and the search terms used [16]. In a recent study of people hospitalized for schizophrenia [17], over 75% of them had used the internet to search mental health–related content in the previous 6 months and individuals “appear to be using the Internet for obtaining information about their early symptoms and experiences prior to their first contact with psychiatric care.”

The apparent frequency and wide-ranging use of the internet by people with psychosis has associated drawbacks, primarily related to the lack of regulation of web-based content. Multiple studies of web-based mental health search behavior among people with psychosis have found that patients express a desire for their mental health clinician to provide a recommended list of reputable websites rather than needing to navigate the plethora of available information unguided [18,19]. Kirschenbaum et al [17] called for more understanding by clinicians of what consumers are looking for when conducting internet searches, and that this increased understanding should help clinicians tailor web-based resources to improve care pathways and reduce the duration of untreated psychosis. Further research has highlighted a need for clear guidance for clinicians aid them in recommending reputable web-based resources to those with severe mental illnesses [20]. As it has been noted that the development of web-based mental health resources and treatments is far outpacing their evaluation [21], mental health professionals would benefit from guidance on what resources to recommend. Hence, it is important that clinicians have some confidence in the quality of information being accessed, and whether it is targeted to the consumers’ needs [22].

Despite the central role that the internet now plays in the delivery of health information and services, clinical services generally do not have formal approaches for using the internet for health information in collaboration with consumers and their support network. Clinicians have limited guidance and time to evaluate the plethora of websites available but are aware that poor-quality information can produce unnecessary worry, increase inappropriate consultations, or lead to use of ineffective treatments [13]. Inconsistencies in the quality and clarity of information on the internet make it difficult for clinicians to help guide patients to the best information available. For example, a meta-analysis [23] revealed that 42% of mental health websites are either owned by, or receive funding from, pharmaceutical companies, and that these websites are significantly more likely to be biased toward recommending medication, and not all websites disclose such funding. It has been noted that aiding clinicians to identify reputable websites has been suggested as an important enhancement to patient care [20]. The challenge for health care professionals is to know how to appraise the trustworthiness of psychosis-related website services to enable them to adequately inform consumers, their families, carers, and the general community [24].

This paper focuses on the clinician’s perspective, with the aim of providing clinicians with a process to aid them in identifying appropriate and reputable websites that may be recommended to people experiencing psychosis and their network with information. This study aimed to (1) identify which websites with psychosis-related content surfaced when common diagnostic terms for psychosis are entered into a popular search engine and (2) rate the quality of these websites using a validated instrument (the DISCERN scale) and a purpose-developed checklist of content defined specifically for psychosis. It is anticipated that these results may be useful for clinicians to guide them in recommending high-quality websites to consumers and families experiencing psychotic disorders.

## Methods

### Measures of Website Quality

This paper uses an operational definition of website “quality” in accordance with the criteria employed by two website quality rating scales: the DISCERN scale [25] and the Psychosis Website Quality Checklist (PWQC) as described below. While the DISCERN scale is the most widely used instrument for evaluating health information websites for any health problem, it does not evaluate specific disorders. The PWQC was used to account for this.

### The DISCERN Scale

The DISCERN tool [25] was developed to “enable patients and information providers to judge the quality of written information about treatment choices.” The tool is freely available in conjunction with a web-based handbook [26] to evaluate internet-based health information. It rates website reliability, treatment options, and the quality of information with 16 items using a 5-point Likert scale across two subscales: Reliability (Is the publication reliable?) and Quality (How good is the quality of information on treatment choices?). The overall DISCERN scale is obtained by summing the first 15 items of the scale (range 15-75). While the DISCERN tool is useful for rendering the results of this study comparable to that of other studies evaluating web-based health information, it is not designed for evaluating the quality of specific disorders or treatment content.

### The PWQC

A disorder-specific website quality checklist was based on the Bipolar Website Quality Checklist (BWQC) devised by Barnes et al [27]. The BWQC has high interrater reliability and a strong correlation with the DISCERN instrument. Five general BWQC subscales were replicated verbatim for the PWQC (see Multimedia Appendix 1):

- Credibility (7 items): is the website reputable, does it have clear quality markers?
- Currency (2 items): is the currency of the website clear?
- Objectivity (6 items): is the website clear about its aims, sponsorship, etc?
- Availability and Usability (4 items): is the website easy to navigate?
- Design and Aesthetics (2 items): are text and images presented in a clear way?

The sixth subscale was adapted from bipolar disorder–specific to schizophrenia-specific content. The Brief Psychiatric Rating Scale [28] and information derived from the Royal Australian and New Zealand College of Psychiatrists Clinical Practice Guidelines for Treatment of Schizophrenia and Related Disorders [29] were used as the basis for developing the 29-item disorder-specific subscale measuring Breadth and Accuracy of the diagnostic and treatment-related information on psychosis.

The individual PWQC items were on a 5-point Likert scale with defined anchor points (1=no, 3=partially, and 5=yes) with a total score ranging 50-250. A PWQC User Guide detailed descriptors of each item and was provided to all raters to enhance interrater reliability (see Multimedia Appendix 1).

### General Website Characteristics

The general website characteristics assessed included reporting the presence of an editorial board, ownership type, and scope of information provided [27,30]. Websites were allocated by 2 authors (KW, CMH) to an organizational type: professional (not-for-profit sites associated with a government or professional body with demonstrated health professional involvement [31]), commercial (associated with a privately owned company, professional individual, or drug company for profit), or consumer (referred to as peer-to-peer online support group, forum, virtual community, social network, bulletin board, web-based discussion forum, or live chat room). Consumer organization websites have been evidenced in previous research to support consumers to feel more active as participants in their health care decisions, improve empowerment, and reduce societal loneliness [32].

### Website Selection

The search terms “psychotic,” “psychosis,” “schizophrenia,” “delusion,” and “hallucination” were entered as a string into the search box for search engine Google. These were selected based on diagnostic terms used by psychiatrists in accordance with the Diagnostic and Statistical Manual of Mental Disorders, fifth edition [33]. Google was selected as the most highly used search engine, with over between 87%-89% of the search market share in the study period. The searches were carried out on a browser where cache, cookies, and browser history were cleared. In line with previous research, the inclusion criterion was that the website fell within the first 25 sites listed by the search engine [34]. Websites were excluded if they were either of the following: a personal blog, news or a media article, not in English, or a paid listing or advertisement. The websites were assessed at 2 time points (January-March 2014 and January-March 2018) by three raters. All identified websites were evaluated using both DISCERN and PWQC instruments. When sites were checked before the second set of assessors in 2018, only one (consumer) site no longer existed and was excluded (see Multimedia Appendix 2 for flowchart and Multimedia Appendix 3 for Results of the Google search of psychosis-related terms).

### Raters

In total, 5 raters evaluated the websites. There were initially 3 raters, but owing to interest expressed by further raters, we repeated the exercise 4 years later. One author (KW) rated at both time points to provide some evaluation of whether the websites returned by the search engine were similar at each time point. However, the second rating was carried out without reference to the previous rating. Raters at each time point included individuals with specialist mental health knowledge and general medical knowledge to ensure that a range of experience and expertise was captured. Thus, raters included a consultant psychiatrist (both time points), and either a clinical nurse consultant or psychiatry registrar and a final year medical student. The aim was to have raters with a capacity to appraise accuracy and quality of the clinical information but with different levels of expertise. All raters read the PWQC manual (see Multimedia Appendix 1) and the DISCERN handbook and evaluated the websites independently of each other. Consumers were not included as the evaluations of content required an ability to critically appraise the clinical information.

### Statistical Analyses

Statistical analysis was conducted using SPSS (version 24; IBM Corp). Each rater’s DISCERN and PWQC scores for each website were calculated as a mean score. For the primary analysis, the Pearson product-moment correlation coefficient was calculated to examine associations between DISCERN and PWQC total scores, and the interrater reliability for each scale was examined using intraclass correlation coefficients and calculated using the 2-way mixed model with consistency type. Two-tailed paired *t* tests were used to determine changes in mean scores on the DISCERN and PWQC instrument between time 1 (2014) and time 2 (2018). One-way analyses of variance were used to compare scores on the DISCERN and PWQC instruments by website ownership type (professional, commercial, or consumer organization).

## Results

### Websites Retrieved

Of the 25 websites described in Multimedia Appendix 3, a total of 9 were rated as commercial organizations (links to pharmaceutical companies or seeking referrals to private services or facilities), 11 as professional organizations (mainly aimed to provide information, often backed by a university or health service rather than relying on advertising or charitable grants), and 5 as consumer organizations. Consumer blogs by people who had lost a close family member or friend to mental illness were not included as they were not intended to provide information about the disorders.

A quality marker was associated with 10 of 25 (40%) websites and membership to a code of conduct with 12 (48%) websites, while 14 (56%) websites had an editorial or review process in place and 21 (84%) websites noted sources of information provided or gave references. There were no significant changes in DISCERN or PWQC scores between the 2 data collection time points. Therefore, data were combined for the remainder of the analyses.

### Overview of the DISCERN Ratings

The mean DISCERN score was 43.96 (SD 12.08) (in Multimedia Appendix 4), which DISCERN scoring criteria categorize as being of “fair” quality (total score 39-50). The range of scores was 32-57, with the 8 lowest-ranking websites being of “poor” quality (total score 27-38) and 6 highest-ranking websites meeting the criteria for “good” quality (total score 51-62). No websites were rated as “excellent.” The mean overall rating (DISCERN item 16) was 2.91 (SD 1.11) of a possible total of 5: the two highest websites received a mean rating of 4.

The intraclass correlation coefficients indicate moderate interrater reliability for Reliability subscale and Overall rating, and good interrater reliability for the Quality subscale and the Total DISCERN score. For each scale, the CIs were wide, suggesting that the true interrater reliability for the Reliability subscale and overall rating was “poor” to “good,” and that of the Quality subscale was “moderate” to “excellent,” and the DISCERN total score was “moderate” to “good.” This reflects variability in the different raters’ appraisal of each website, suggesting that while there were differences in the perception of each website’s reliability, clarity, and sources of information, there was greater rater agreement on the quality of information provided.

### Overview of the PWQC Ratings

The overall mean score on the PWQC was 147 of a possible 250 points (Multimedia Appendix 5). Websites were rated most highly on the “Availability and Usability” subscale, where on average, websites achieved 84% of highest possible score (16.82 of possible 20), followed by “Design and Aesthetics” and “Objectivity” subscales, achieving 70% and 64% (7/10 and 19/30) of total possible scores, respectively. Websites performed poorly on remaining subscales, achieving 51%-59% of the total possible score for “Credibility,” “Currency,” and “Breadth and Accuracy” (21/35, 5/10, and 78/145, respectively).

The intraclass correlation coefficients indicate that the interrater reliability for most subscales was “moderate” (0.50-0.75). The “Breadth and Accuracy” subscale achieved “good” interrater reliability, while the interrater reliability for “Availability and Usability” was “poor.” However, considering the wide confidence intervals, the true interrater reliability for most subscales is likely to be in the range of “poor” to “good.”

The mean scores for each PWQC subscale and the total DISCERN score were grouped in accordance with organization type of the site, as proposed by Griffiths and Christensen [30]. There was no difference in website quality by organization category, except for the “Design and Aesthetics” subscale, which revealed a significantly poorer rating for commercial sites than for professional or consumer sites (Multimedia Appendix 6). Websites with an editorial board or review process were rated significantly more highly on overall PWQC and DISCERN scales, and on Credibility and Currency subscales.

There was room for comments on the rating sheet and these included notes on the lack of mention of suicide, other risks, and lack of information about the importance of early intervention, particularly for first episodes.

### Correlations Between the DISCERN and PWQC Instruments

The relationship between the mean total DISCERN and PWQC scores was investigated using the Pearson product-moment correlation coefficient. Preliminary analyses were performed to ensure that no violation of assumptions of normality, linearity, and homoscedasticity was observed in the 2 scores. There was a strong positive correlation between the DISCERN and PWQC variables (*r*=0.85, *P*<.001). Indeed, although the top-ranking website on each scale was different, 9 of the top 10 websites on each scale were the same.

## Discussion

### Principal Findings

This paper aimed to identify and explore the quality of websites with psychosis-related content found through a common search engine. While psychosis-related websites were generally easy to identify using common search terms, the quality of websites was overall poor to moderate, with little evidence of change in quality over a 4-year period.

### Quality of Websites

Relevant websites were easy to identify with a common search engine using general search terms related to psychosis, and the search results were consistent over time. The “fair” performance of websites in this study is largely consistent with that reported in previous research examining website quality related to other mental disorders. When developing the BWQC, Barnes et al [27] reported that the 15 websites they evaluated were “disappointing” in their performance on both the BWQC and DISCERN scales. Similarly, Nemoto et al [35] evaluated 37 websites with the DISCERN scale, mostly focusing on mood disorders, panic disorder, and schizophrenia, and concluded that the information provided was generally inadequate, with an overall mean score of 46 of 75. More recently, Rathod, et al [36] reported that only 8 of 27 depression websites focusing on depression scored well on the DISCERN scale, while an evaluation of 20 websites on perinatal anxiety Kirby et al [37] reported that all websites were ranked low to moderate on the DISCERN scale. The Health of Nations Code provides an ethical code for websites and is based on eight principles, which are included in the DISCERN scale categories [38]: authority, complementarity, privacy policy, attribution and date, justifiability, transparency, financial disclosure, and advertising policy. 

### Findings Related to Specific Psychosis-Related Topics

Another finding was the generic nature of the information available on the websites and the lack of information on areas associated with controversy, such as recreational drug use or coercive treatment. The risks and benefits of medication were not detailed, nor were the importance of physical health aspects of treatment.

The assessors had different levels of experience (by design), and the greater agreement over quality of the website than the actual content is consistent with this observation. While some websites mentioned families and carers, most websites did not provide families with much information that would be potentially helpful in their interactions with the family member experiencing psychosis or treating clinicians. Although avoiding contentious areas may be understandable, these areas may be precisely the topics that consumers and carers want to learn more about for themselves, and we suggest that websites could at least provide a series of questions for consumers and carers to ask the treating clinicians. More recently, some US clinical groups have produced lists of question prompt lists for consumers and their families in relevant areas (and are available through Google), which seems to be a productive initiative [39].

While people with psychosis are seeking web-based information, which can be helpful as supplementary information in collaborative planning with clinicians [18], few websites mentioned the importance of early intervention or approaches to maximize recovery and long-term outcomes. These approaches are now established practice in the treatment of psychosis by mental health services in Australia [3]. We found that the same websites tended to perform relatively well on both the DISCERN and PWQC scales, although the DISCERN scale is a general measure of health information websites, and the PWQC was more psychosis specific. This enabled us to produce a relatively consistent “top 10” websites that clinicians may recommend to consumers and families with a psychosis-related condition. This is consistent with the findings of Barnes et al [27] when evaluating depression-related websites. The better-performing websites tended to display an editorial board or review process, regardless of website ownership type, suggesting that these content control measures are better indicators of website quality than organization ownership. These sites tended to belong to professional organizations associated with a government or professional body, and professional websites have previously been found to contain better-quality information on other conditions such as depression in previous research [31]. There have been mixed findings regarding the importance of website ownership type, with some studies finding that websites owned by professional or charitable organizations were of higher quality [40] and others revealed no effect [41]. It is important to note that categorizing websites was not a straightforward process when allocating to just one category. For example, many nonprofit websites still contained paid advertising and links to commercial products. As web-based advertising increases and the internet continues to evolve, these categories of ownership type may be less meaningful than that when originally conceived.

Of particular concern were our findings that website quality did not improve much over time. Although there were some nonsignificant improvements in the presentation of information on the sites, there was little change in the quality of the content of psychosis-related websites over the 4-year period of the study. This is consistent with the findings of Walsh et al [42], who reported that almost half of depression websites did not update their content over a period of 7 years, prompting them to conclude that “the internet is used more than it is trusted.” Considering the importance of the internet as a source of information for people with a mental illness, as well as for their friends and family, it is concerning that many websites do not appear to provide current or comprehensive information and tend not to address issues such as early symptoms of psychosis and suicide risk, which are topics that consumers may be seeking, particularly early in their condition [17], but we have sought to identify the “best available” websites for clinicians to recommend in clinical conversation about psychotic disorders.

### Limitations

Although most Australians speak English, the researchers recognize the ever-increasing diversity within the Australian population and worldwide. The inclusion of only English-language psychosis websites within this paper is a limitation, with research of websites in other languages an area of future need. Secondly, the selection of raters from a health professional background was intentional but meant that there was no consumer involvement. We intend to further investigate the process by which consumers and carers assess and judge the quality of health-related internet-based information. Finally, the interrater reliability on some subscales of the DISCERN and PWQC instruments was low, which may have influenced the accuracy of the results. Anecdotally, a lot of the variability between raters came from the ease of navigating the site, with some raters reporting that some websites required a high number of “clicks” through various tabs or pages to find the desired content.

### Conclusions

The internet can provide clinicians with information that can improve decision-making with consumers and their families, but accessing helpful information can be overwhelming. However, most sites generally avoid contentious areas related to addressing illicit drug use, weighing up risks and benefits of medication or the role of coercive treatment. Insufficient emphasis is placed on providing detailed information on early intervention and the importance of lifestyle modifications, such as exercise programs or how families and friends can contribute. This is likely to be the very information that consumers and carers are seeking and thus contributes to the unmet needs of this group.

While higher-quality websites exist, there is generally no easy way to assess this on face value, as common markers such as website ownership type are not always associated with the breadth or reliability of information available. Generally, sites providing evidence of their editorial process were the most helpful. There remains significant room for improvement in website quality; however, through our review process, we were able to rank websites consistently on 2 quality scales, thereby producing a resource that may guide professionals when recommending web-based resources to consumers and carers. Our findings indicate a need for health care providers and government agencies to address the issues of poor quality, keeping information current and providing prompts about how to address more controversial areas to be available to consumers and carers.

## Appendix

Multimedia Appendix 1 The Psychosis Website Rating Scale for first 3 websites (as example) and rating instruction manual.

Multimedia Appendix 2 Flowchart of inclusion and exclusion of websites.

Multimedia Appendix 3 Results of the Google search of psychosis-related terms.

Multimedia Appendix 4 Ranking of mean (SD) scores for identified websites by total DISCERN scale, Reliability and Quality subscales.

Multimedia Appendix 5 Mean (SD) total scores on the PWQC tool and subscales.

Multimedia Appendix 6

## Abbreviations

BWQC: Bipolar Website Quality Checklist
PWQC: Psychosis Website Quality Checklist
