# Supplementary material for: The Quality of Internet Websites for People Experiencing Psychosis: Pilot Expert Assessment
Source: JMIR Form Res. 2022 Apr 15;6(4):e28135. doi: 10.2196/28135 (PMC9055477; doi:10.2196/28135)
Supplement: Multimedia Appendix 1 [file formative_v6i4e28135_app1.pdf]

The Psychosis Website Rating Scale for first 3 websites and rating instruction manual

Please read the *Guidelines for Psychosis Websites Quality Ratings* before you begin.
Use the rating scale below to rate each website, by writing a number from 1 to 5 next to each item.

RATING SCALE	1	2	3	4	5
	No		Partially		Yes

Reviewer Name		A1	A2	A3
	UIN	A1	A2	A3
	Date			
	Website	Reach Out	Brain and Behaviour Research Foundation	About health
	URL	http://au.reachout.com/all-about-psychosis	https://bbrfoundation.org/schizophrenia	http://bipolar.about.com/od/psychoticfeatures/
	No pages/clicks			

		A1	A2	A3
**PART I: Psychosis Quality Website Checklist**				
1. Credibility:				
1.1	In your view does the URL indicate reputable affiliation?			
1.2	Is there a quality marker associated with the website?			
1.3	Does the website show affiliation to code of conduct for their organisation			
1.4	Is there contact information available for website?			
1.5	Is there an overall editorial/ review process in place?			
1.6	Are sources of information/ references shown?			
1.7	Are the credentials of authors shown on the webpage or in 'about us'?			
2.Currency				
2.1	Is the date of website creation shown?			
2.2	Are the pages stamped with date of last update?			
3. Objectivity				
3.1	Are aims of the site clearly stated?			
3.2	Is the intended audience made clear?			
3.3	Is there disclosure of sponsorship/funding source or conflict of interest?			
3.4	Is there advertising on the same pages as factual information is presented?			
3.5	Is the information presented evidence-based?			
3.6	Are areas of debate/uncertainty discussed?			
4. Availability and Usability				
4.1	Site contains current external links to other sites?			
4.2	Search tools present in website			
4.3	Navigation tools/internal links allow easy access around site/ return to homepage			
4.4	Information on psychosis readily found from the landing page			
5. Design and Aesthetics				
5.1	Text clear and presented in easy to read sections			
5.2	Images used in a manner that enhances use of site			
6. Breadth and Accuracy: Does this website provide information on				
6.1	Definition of Psychosis or Schizophrenia			
6.2	Aetiology/cause			
6.3	Associated DSM- disorders			
6.4	Common comorbidities			
6.5	Hallucinations			
6.6	Delusions			
6.7	Unusual Motor Behaviour			
6.8	Negative symptoms			
6.9	Cognitive disturbance			
6.10	Affective disturbance			

6.11	Clinical High Risk			
6.12	Potential early warning signs and early intervention			
6.13	First Episode Psychosis			
6.14	Seriousness of disorder and risk of suicide			
6.15	Pharmacological interventions			
6.16	Risks/side effects of pharmacological intervention			

**RATING SCALE**	**1**	**2**	**3**	**4**	**5**
	**No**		**Partially**		**Yes**

	Website	Reach Out	Brain and Behaviour Research Foundation	About health
	URL	http://au.reachout.com/all-about-psychosis	https://bbrfoundation.org/schizophrenia	http://bipolar.about.com/od/psychoticfeatures/
	**Psychosis Quality Website Checklist continued**			
6.17	Different types of treatment for acute episode and maintenance treatment			
6.18	Role of lifestyle in staying well			
6.19	Psychosocial interventions			
6.20	Family Interventions			
6.21	Psychoeducation			
6.22	Cognitive Behavioural Therapy			
6.23	Social Skills Training			
6.24	Vocational rehabilitation			
6.25	Mental Health Act and Involuntary treatment			
6.26	Biological and psychological treatments both described as being effective and important			
6.27	Substance abuse			
6.28	Self help groups			
6.29	Strengths and Recovery			

**PART II DISCERN**				
1. Is the publication reliable?				
**1**	Are the aims clear? (If No skip to Q3)			
**2**	Does it achieve its aims?			
**3**	Is it relevant?			
**4**	Is it clear what sources of information were used to compile the publication (other than the author or producer)?			
**5**	Is it clear when the information used or reported in the publication was produced?			
**6**	Is it balanced and unbiased?			
**7**	Does it provide details of additional sources of support and information?			
**8**	Does it refer to areas of uncertainty?			
2. How good is the quality of information on treatment choices?				
**9**	Does it describe how each treatment works?			
**10**	Does it describe the benefits of each treatment?			
**11**	Does it describe the risks of each treatment?			
**12**	Does it describe what would happen if no treatment is used?			
**13**	Does it describe how the treatment choices affect overall quality of life?			
**14**	Is it clear that there may be more than one possible treatment choice?			
**15**	Does it provide support for shared decision-making?			
3. Overall Rating of the Publication				
**16**	Based on the answers to all of the above questions, rate the overall quality of the publication as a source of information about treatment choices using the scale below			

**1**	**2**	**3**	**4**	**5**

Low		Moderate		High
Serious or extensive shortcomings		Potentially important but not serious shortcomings		Minimal shortcomings
**COMMENTS**				

FACES IN THE STREET, ST VINCENT'S HOSPITAL

Guidelines for Psychosis Websites Quality Ratings

A User Guide for Rating Websites

Written by Lisa Robins, Joanna Crawford, Therese Fletcher, Victoria Malone

2014

Table of Contents

Overall Rating Instructions

The Rating Scales

PART I: Psychosis Quality Website Checklist Instructions

PART 2: DISCERN Scale

Introduction

Welcome to the Guidelines for Psychosis Websites Quality Ratings.
These guidelines are to accompany the Rating Form: Psychosis Websites Quality Ratings Study being conducted by Faces in the Street: Urban Mental Health Research Institute, St Vincent's Hospital, Sydney.
The aim of this study is to review the quality of commonly found websites on psychosis. It involves several independent raters assessing websites according to the Rating Form: Psychosis Websites Quality Ratings Study.
There are two checklists included in the Rating Form: Psychosis Websites Quality Ratings Study:

i) **Psychosis Quality Website Checklist**
The 29-item Psychosis Quality Checklist was developed as part of this study. Guidelines for completing it are including in this document.

ii) **DISCERN**
The 16-item DISCERN validated tool was developed to assist in the evaluation of all health websites. Guidelines for completing it are provided in the separate document The DISCERN Handbook.

These guidelines aim to assist raters in completing the Rating Form: Psychosis Websites Quality Ratings Study and to streamline the process of rating common psychosis websites according to the criteria provided.

These guidelines provide both overall rating instructions and detailed instructions for each of the individual items of the 29-item Psychosis Quality Website Checklist.
They were developed by the authors with reference to the literature on health website evaluation and psychotic disorders, in addition to their clinical experience.

Overall Rating Instructions

Before commencing rating, please ensure that you have three documents available to refer to:

1) Rating Form: Psychosis Websites Quality Ratings Study
2) Guidelines for Psychosis Websites Quality Ratings (this document)
3) The DISCERN Handbook

Rating Form: Psychosis Websites Quality Ratings Study

You may find it easiest to have the Rating Form printed out, and to handwrite in your ratings as you refer to the websites, prior to typing them in.
On each page of the Rating Form there will be three websites to be rated, each with their own column. **The URL of the website to be rated is listed at the top of each column. The column for rating for each website continues over 2 pages.** The 2-page Rating Form is repeated 9 times (with the URLs for the different websites listed), resulting in an 18-page document.

Websites to be rated

There are 25 websites to be rated.
Note that the URL for each website provided on the Rating Form may not be the homepage for that website, but will be the 'landing page' that was obtained in our search strategy for common psychosis websites. For example, you may be provided with the URL 'au.reachout.com/all-about-psychosis', not the homepage for that website 'au.reachout.com'.

When you rate the information provided by each website according to checklist items on the Rating Form, note that you will be rating the information provided by that **website overall, not just the landing page.** (The

landing page is the page you land on once you enter the URL provided.) This may mean that you will have to click on different pages of the website in order to find the relevant information.

Steps for rating websites

Please rate each website one at a time.
For each website that you rate, refer to the column for that website on your Rating Form.
The URL of the website to be rated is listed at the top of each column. Enter that URL in your computer browser, to access the website.
DATE: For each website that you rate, please write the date in the space provided.

RATE: You are now ready to rate that website according to the Rating Scale.

The Rating Scale

Questions on the Rating Form, for both PART I: Psychosis Quality Website Checklist and

PART II: DISCERN items are all rated on a 5-point scale ranging from No to Yes.

Please use the rating scale below to rate each checklist item on Rating Form, for each website. Go to the URL for the website in that column and rate by entering a number according to the rating scale. Enter one number in each cell using the rating scale below.

Enter only one number from 1 to 5 for each item.

1	2	3	4	5
**No**		**Partially**		**Yes**

The rating scale has been designed to help you decide whether the quality criterion in question is present or has been 'fulfilled' by the information provided in the website. General guidelines are as follows:

- **5** should be given if your answer to the question is a definite 'yes' - the quality criterion has been completely fulfilled
- Partially **(2-4)** should be given if you feel the website being considered meets the criterion in question to some extent. How high or low you rate 'partially' will depend on your judgement of the extent of these shortcomings
- **1** should be given if the answer to the question is a definite 'no' - the quality criterion has not been fulfilled at all

(The only item not rated according to this scale is the last Item 16 on the DISCERN, which has its own rating scale provided.)

PART I: Psychosis Quality Website Checklist Instructions

PQWC has six sections containing 29 quality website criteria items.

Please use the rating scale of 1-5 on previous page to rate each item from web addresses.

nstructions for Specific Items of the Psychosis Quality Website Checklist

In order to assist raters in rating some specific items, the following table provides more detail on the website features and clinical content on the websites being assessed.

1.2	Is there a quality marker associated with the website?	A 'quality marker' refers to a certification of quality displayed on the web site. An example would be<br>HONcode
4.4	Information on psychosis readily found from the landing page	The 'landing page' is the page you land on once you enter the URL provided
6.1	Definition of Psychosis or Schizophrenia	Website provides a definition:<br>Psychosis is not a diagnosis but a symptom or set of symptoms that can have many different causes. Psychosis may be transient, intermittent, short term or part of a longer term psychiatric condition. Psychosis is defined as a loss of contact with reality, typically marked by delusions and/or hallucinations
6.2	Aetiology/cause	The following risk factors should be mentioned:<br>1. Genetic vulnerability, eg, family history of psychosis<br>2. Neurodevelopmental vulnerability, eg perinatal birth complications and in utero viral exposure.<br>3. Extrinsic factors, eg substance abuse, viral infections and developmental stress<br>4. Gender, eg different rates, duration, severity.<br>5. Urban/rural differencces.<br>6. Substance use.
6.3	Associated DSM-disorders	Associated DSM-IV diagnoses include:<br>1. Schizophreniform disorder; 2. Schizophrenia; 3. Schizoaffective disorder; 4. Bipolar mood disorder; 5. Unipolar psychotic depression 6. Delusional disorder; 7. Psychosis due to general medical conditions 8. Substance-induced psychosis
6.4	Common comorbidities	The following should be outlined:<br>1. Substance use<br>2. Anxiety<br>3. Depression<br>4. Physical health and metabolic syndrome
6.5	Hallucinations	Description of the following forms of hallucination is present:<br>1. Auditory. Hearing voices in the absence of auditory stimulus.<br>2. Tactile. Sensing things by touch in the absence of tactile stimulus.<br>3. Visual. Seeing things in the absence of visual stimulus<br>4. Olfactory. Smelling things in the absence of olfactory stimulus<br>5. Gustatory. Tasting things in the absence of gustatory stimulus.

		6. Command. Hearing and sometimes obeying voices that command them to perform certain acts. The hallucinations may influence them to engage in behaviour that is dangerous to themselves or to others.
6.6	Delusions	Descriptions of the following forms of delusion is present:<br>1. Grandiosity. Exaggerated self-opinion, conviction of special abilities or powers or identity such as someone rich or famous.<br>2. Persecutions delusions and paranoia. Expressed or apparent belief that other persons have acted maliciously or with discriminatory intent. Includes persecution by supernatural or other non-human agencies (eg, the devil).<br>3. Unusual, odd, strange, bizarre thought content. Delusions are clearly false/bizarre ideas expressed with full conviction.<br>4. Ideas/delusions of reference. Beliefs that casual events, people's remarks, etc. are referring to oneself when, in fact, they are not.
6.7	Unusual Motor Behaviour	The following unusual motor behaviours should be outlined:<br>1. Abnormality of movements not associated with a medical illness<br>2. Physical tension, overactivity (eg, pacing) or aggression<br>3. Bizarre or disorganised behaviour such as posturing or catatonia
6.8	Negative symptoms	The following should be outlined:<br>1. Flattened affect; 2. Poverty of thought; 3. Lack of motivation<br>4. Social withdrawal; 5. Anhedonia
6.9	Cognitive disturbance	The following cognitive disturbances should be outlined:<br>1. Impaired working memory, verbal memory<br>2. Impaired executive function<br>3. Distractibility / inattention
6.10	Affective disturbance	The following affective disturbances should be outlined:<br>1. Elevation (mania); 2. Anxiety; 3. Depression/suicidal<br>4. Aggression/hostility
6.11	Clinical high risk	1. Websites provides information on groups at high risk of developing psychosis: The ultra-high or at risk mental state involves:<br>a) Young people, usually aged 14 and 35 years.<br>b) Change in subjective experience/behaviour in recent months or within past 5 years (may fluctuate but is persistent, often progressive).<br>c) EITHER subthreshold positive symptoms (not severe/persistent enough to be evidence of sustained frank psychosis sufficient for a Dx of DSM psychotic disorder - other than brief psychosis, OR a family history of psychotic disorder or schizotypal disorder in first degree relative + significant yet nonspecific decline in psychosocial functioning within past year or which is not resolving.<br>2. Website notes that ‘prodrome’ (prolonged period of symptoms, increasing disability) can only be acknowledged retrospectively.
6.12	Potential early warning signs	Website provides information about potential early warning signs of psychosis, including:<br>a) Worrisome drop in grades or job performance;

	and early intervention	b) New trouble thinking clearly or concentrating;<br>c) Suspiciousness/uneasiness with others;<br>d) Decline in self-care or personal hygiene;<br>e) Spending a lot more time alone than usual;<br>f) Increased sensitivity to sights or sounds;<br>g) Mistaking noises for voices;<br>h) Unusual or overly intense new ideas; and<br>I) Strange new feelings or having no feelings at all.<br>Website promotes importance of early identification and evaluation of the onset of psychosis as an important health concern, and that outcomes are improved with earlier detection and intervention.
6.13	First Episode Psychosis	Website explains that a first episode of psychosis is the first time a person experiences a psychotic episode. Provides psychoeducation around experience of a first episode and the importance of seeking treatment.
6.14	Risk of suicide	Website acknowledges the seriousness of disorder and risk of suicide
6.15	Pharmacological interventions	The following interventions should be described:<br>1. Antipsychotics, including Depot medication<br>3. Anti-anxiety and antidepressant medication<br>4. Mood-stabilisers
6.16	Risks/side effects of pharmacological intervention	1. Metabolic effects – weight gain, diabetes, increased lipids levels<br>2. Tardive Dyskinesia and extra perinatal side effects<br>3. Polysubstance use<br>4. Pregnancy and breastfeeding
6.17	Different types of treatment for related to stages/phases	Website provides information on acute and maintenance treatment: Management of schizophrenia is best considered in stages/phases (1st episode psychosis, recurrent/persistent psychosis including treatment of relapse/relapse-prevention, maintenance, treatment refractory SZ.
6.18	Role of lifestyle in staying well	1. The importance of employment vocation as a meaningful activity<br>2. Social contact<br>3. Physical health (Nutrition, exercise and recreational activities)<br>4. Substance use (smoking and use of other illicit drugs or prescription drugs for non-prescription purposes)
6.19	Psychosocial interventions	Website promotes use of psychosocial interventions relevant to their needs, informed by understanding of social and cultural context.
6.20	Family Interventions	Website promotes use of family interventions where possible and appropriate. Families inevitably experience substantial psychological and social effects themselves following the onset and during the course of schizophrenia. Families benefit from an intervention that offers knowledge about the illness, its effects on affected person and family, and provides an avenue of support and assistance for them.
6.21	Psychoeducation PE	Website promotes use of PE and concept they improve treatment adherence, outcomes, better Mx of relapse, lower readmission rates, greater well-being. Early phase PE focuses on supporting/educating patient and/or family about illness, using biopsychosocial perspective.

		Later phase PE may evolve to other topics, eg, life skills, adapting to changes necessary to manage their illness.
6.22	Cognitive Behavioural Therapy CBT	Website promotes use of CBT especially when positive symptoms are slow to respond/refractory to drug treatment, may also target distress and comorbidity.
6.23	Social Skills Training SST	Website promotes use of SST to improves social adjustment, enlarges and enhances the person's social network and contributes to the development of independent living skills.
6.24	Vocational rehabilitationVR	Website promotes concept that VR likely to have positive psycho-social consequences. There are two main models of VR: prevocational training (period of preparation prior to seeking competitive employment) and supported employment (people are placed in competitive employment with provision of on-the job support). The IPS model of cognitive remediation is emphasised (computer tasks to reverse cognitive deficits).
6.25	Mental Health Act, Involuntary treatment	Website gives a laymen's explanation of why involuntary treatment comes about and what is involved
6.26	Biological and psychological treatments	Website acknowledges importance of biological and psychological treatments.
6.27	Substance abuse	1. Website provides psychoeducation about interaction effect and exacerbation of psychosis with substance use: In general, the younger the person is, the greater the risk posed by the use of substances.<br>2. Website lists substances known to have links to possible psychosis:<br>a) Marijuana/hash/THC ; b) Methamphetamine (including crystal meth) ; c) PCP/Psilocybin/Peyote /Mescaline ; d) LSD ; e) Ketamine<br>f) Steroids ; g) Amphetamines/stimulants
6.28	Self-help groups	1. The benefits of self-help groups<br>2. Format of self-help groups<br>3. Contacts of how to find local self-help groups
6.29	Strengths and Recovery	Recovery from SZ occurs more frequently than once believed, many go on to enjoy a full and meaningful life. The website should discuss:<br>a) concept of recovery<br>b) promote the identification of personal strengths

PART II: DISCERN Instructions

Instructions for completing the DISCERN provided separately in 'The DISCERN Handbook'. Please refer to this when completing the PART II: DISCERN in Rating Form.

General Instructions for DISCERN

DISCERN has three sections containing 16 quality website criterions items in total.
DISCERN consists of 15 key questions plus an overall quality rating. Each question represents a separate quality criterion (an essential feature or standard that is an important part of good quality information on treatment choices).
The DISCERN Questions are organised in three sections as follows:

- Questions 1-8 address reliability of the publication and should help you consider whether it can be trusted as a source of information about treatment choices
- Questions 9-15 focus on specific details of information about treatment choices. NB: apart from Q14, the questions are concerned with the treatment choice(s) described in the publication, and not with all possible treatment choices. Qs 9-11 are concerned with the 'active' treatments described in the publication and can include self-care. 'No treatment' options are dealt with separately in Question 12
- Occasionally, a question is not appropriate for a publication. Eg, the question about 'no treatment' options would not be appropriate for a publication about labour and birth. You should use your judgement to exclude a question that is not relevant. However, DISCERN has been developed as an appraisal process and should be used in its entirety. You must not use individual questions or sets of questions separately. You will find it easiest to read the publication fully before answering the DISCERN questions.
- Q16 is the overall quality rating at the end of the instrument. Your answer to this question should be based on your judgement of the quality of the publication as a source of information about treatment choices after rating each of the 15 preceding questions. However, you should only rate a publication as good quality if it rated well on the majority of questions. Question 16 on the DISCERN uses a different rating scale to the other items. ***Please use the below rating scale to rate item 16***

**1**	**2**	**3**	**4**	**5**
**Low**		**Moderate**		**High**
Serious or extensive shortcomings		Potentially important but not serious shortcomings		Minimal shortcomings

Contact

If you have any queries about rating websites for the Psychosis Websites Quality Ratings Study, please contact Joanna Crawford (Research Officer) on:
Ph: 8382 1660 E: Joanna.Crawford@svha.org.au

References

Charnock D. *The DISCERN Handbook:Quality criteria for consumer health information on treatment choices.* Oxon, United Kingdom: : Radcliffe Medical Press Ltd; 1998.
McGorry P, Killackey E, Elkins K, Lambert M, Lambert T. Summary Australian and New Zealand clinical practice guideline for the treatment of schizophrenia (2003). *Australasian Psychiatry.* 2003;11(2):136-147
