# Supplementary figures and images for: The Quality of Internet Websites for People Experiencing Psychosis: Pilot Expert Assessment
Source: JMIR Form Res. 2022 Apr 15;6(4):e28135. doi: 10.2196/28135 (PMC9055477; doi:10.2196/28135)

Flow diagram of inclusion and exclusion of websites for evaluation

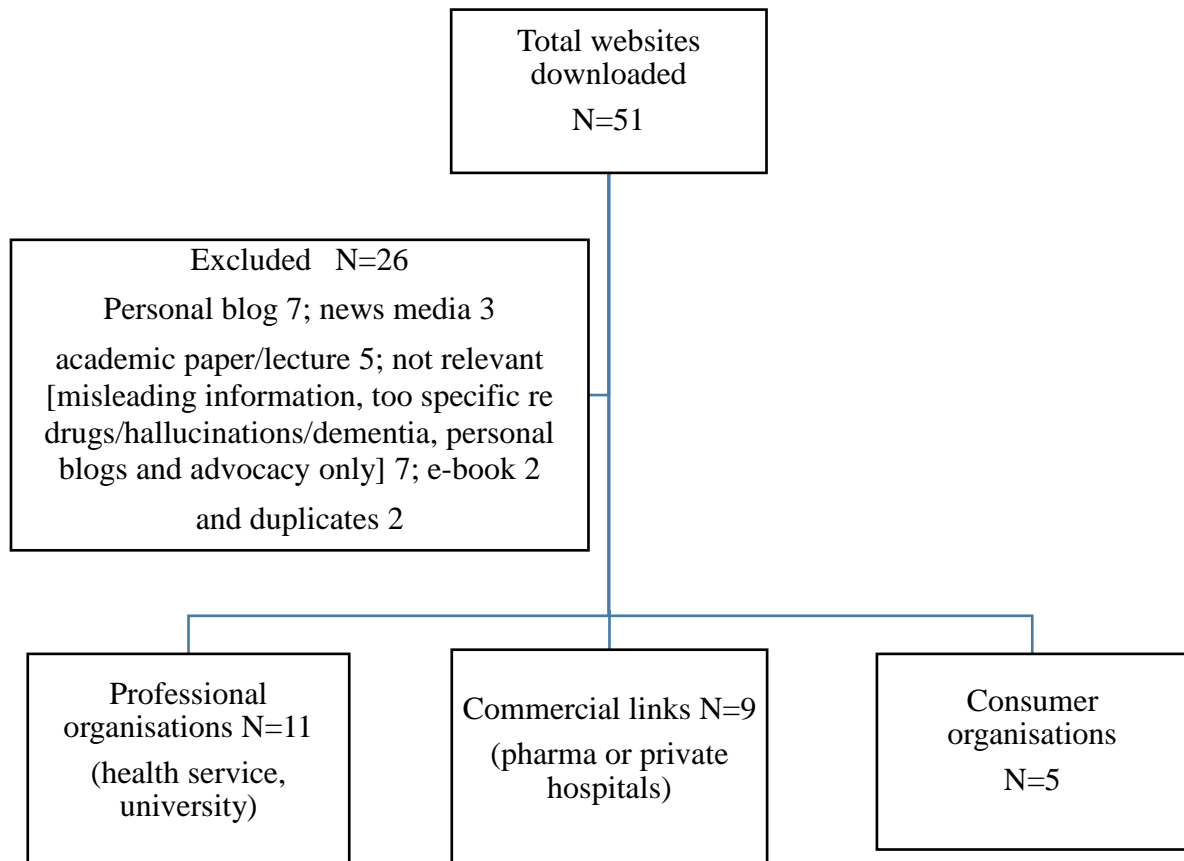

Supplement: Multimedia Appendix 2 [file formative_v6i4e28135_app2.pdf]
