# Supplementary material for: The Quality of Internet Websites for People Experiencing Psychosis: Pilot Expert Assessment
Source: JMIR Form Res. 2022 Apr 15;6(4):e28135. doi: 10.2196/28135 (PMC9055477; doi:10.2196/28135)
Supplement: Multimedia Appendix 3 [file formative_v6i4e28135_app3.pdf]

Results of the Google search of psychosis-related terms

Search result	Website	URL	Organisation type	[a]Website reputation information
1	Reach Out	http://au.reachout.com/all-about-psychosis	Professional	EB
2	Brain & Behaviour Research Foundation	https://bbrfoundation.org/schizophrenia	Professional	Nil
3	About Health	http://bipolar.about.com/od/psychoticfeatures/	Commercial	Nil
4	Wikipedia	http://en.wikipedia.org/wiki/Psychosis	Consumer	EB
5	Early Psychosis Prevention and Intervention Centre	http://eppic.org.au/psychosis	Professional	Nil
6	Free Dictionary by Farlex	http://medical-dictionary.thefreedictionary.com/psychosis	Commercial	Nil
7	Orygen Youth Health Clinical Program	http://oyh.org.au/our-services/training-resources/free-downloads-youth-mental-health-resources/fact-sheets	Professional	Nil
8	Psych Central/ Psychosis_explained	http://psychcentral.com/disorders/schizophrenia/	Commercial	QM, CC, EB
9	Schizophrenia.com	http://schizophrenia.com/	Consumer	CC, EB
10	Better Health Channel	http://www.betterhealth.vic.gov.au/bhcv2/bhcarticles.nsf/pages/	Professional	EB
11	Headspace	www.headspace.org.au/is-it-just-me/find-information/psychosis	Professional	Nil
12	Healthline	http://www.healthline.com/health/psychosis#Overview1	Commercial	QM, CC, EB
13	HelpGuide	http://www.helpguide.org/home-pages/schizophrenia.htm	Consumer	EB
14	Mayo Clinic	http://www.mayoclinic.org/diseases-conditions/schizophrenia/basics/definition/con-20021077?reDate=23112014	Professional	QM, CC
15	Medical News Today	www.medicalnewstoday.com/articles/248159	Commercial	QM, CC, EB
16	Mental Health America	http://www.mentalhealthamerica.net/conditions/schizophrenia	Consumer	Nil
17	MedicineNet	www.medicinenet.com/psychotic_disorders/article.htm	Commercial	QM, EB
18	MentalHelp.net	www.mentalhelp.net/poc/center_index.php?id=7	Commercial	Nil
19	News-Medical.net	www.news-medical.net/health/Psychosis-What-is-Psychosis.aspx	Commercial	CC
20	National Health Service (UK)	http://www.nhs.uk/conditions/Psychosis/Pages/Introduction.aspx	Professional	QM, CC, EB

21	National Institute of Mental Health	www.nimh.nih.gov/health/topics/schizophrenia	Professional	CC, EB
22	Medline Plus	www.nlm.nih.gov/medlineplus/ency/article/001553.htm	Professional	QM, CC, EB
23	Patient.co.uk	www.patient.co.uk/health/schizophrenia-leaflet	Professional	QM, CC, EB
24	Sane	www.sane.org/information/factsheets-podcasts/185-psychosis	Consumer	QM, CC
25	Web MD	www.webmd.com/schizophrenia/	Commercial	QM, CC, EB

[a]QM = quality marker, CC = code of conduct, EB = editorial or review board, Nil = no apparent quality controls
