# Supplementary material for: The Quality of Internet Websites for People Experiencing Psychosis: Pilot Expert Assessment
Source: JMIR Form Res. 2022 Apr 15;6(4):e28135. doi: 10.2196/28135 (PMC9055477; doi:10.2196/28135)
Supplement: Multimedia Appendix 4 [file formative_v6i4e28135_app4.pdf]

Ranking of mean (SD) scores for identified websites by total DISCERN scale, Reliability and Quality subscales

Website	Reliability	Quality	Overall rating	Total DISCERN
Schizophrenia.com	32.17 (9.02)	25.78 (7.19)	4.00 (1.10)	57.94 (16.09)
Patient.co.uk	33.50 (4.42)	23.17 (4.49)	3.60 (0.55)	56.67 (6.71)
NHS	31.33 (4.37)	23.33 (3.88)	4.00 (1.00)	54.67 (7.79)
MedicineNet	29.67 (9.18)	24.17 (5.64)	3.60 (0.55)	53.83 (14.55)
Early Psychosis Prevention and Intervention Centre	31.60 (5.18)	21.40 (7.71)	3.60 (0.55)	53.00 (9.46)
HelpGuide	29.67 (3.50)	23.00 (6.57)	3.83 (1.17)	52.67 (9.22)
National Institute of Mental Health	28.00 (6.48)	22.00 (6.78)	3.80 (1.10)	50.00 (10.55)
Psych Central	27.00 (9.38)	22.17 (10.11)	3.33 (1.51)	49.17 (18.63)
Mentalhelp.net	26.00 (9.93)	22.00 (5.59)	3.40 (0.55)	48.00 (12.46)
Wikipedia	32.07 (3.97)	15.17 (3.31)	3.08 (0.66)	47.24 (3.22)
WebMD	26.33 (7.81)	20.00 (5.62)	3.50 (1.38)	46.33 (12.47)
Orygen Youth Health Clinical Program	24.83 (5.56)	18.50 (3.45)	3.00 (0.89)	43.33 (8.31)
SANE	27.17 (4.75)	15.17 (5.95)	2.83 (1.17)	42.33 (9.93)
Mayo Clinic	24.98 (7.20)	15.83 (2.40)	2.67 (0.52)	40.81 (9.32)
Medical News Today	27.00 (7.56)	12.33 (3.67)	2.20 (1.10)	39.33 (7.00)
About Health	21.20 (5.26)	17.40 (9.56)	2.40 (1.14)	38.60 (13.99)
Better Health Channel	25.24 (4.33)	13.00 (3.29)	2.17 (0.98)	38.24 (6.97)
New-Medical.net	22.00 (7.48)	15.67 (2.07)	2.20 (0.84)	37.67 (7.47)
Mental Health America	22.17 (5.19)	15.50 (5.28)	2.50 (0.84)	37.67 (8.80)
Medline Plus	26.50 (4.51)	11.00 (3.16)	2.00 (1.00)	37.50 (5.96)
Headspace	26.29 (4.47)	10.83 (4.07)	2.50 (1.22)	37.12 (5.93)
Free Dictionary by Farlex	24.19 (6.55)	12.08 (5.33)	2.08 (0.49)	36.27 (11.43)
Healthline	21.83 (8.04)	13.33 (3.14)	2.17 (0.75)	35.17 (7.17)
Reach Out	22.50 (2.88)	10.83 (4.02)	2.50 (1.22)	33.33 (6.12)
Brain & Behaviour Research Foundation	22.33 (5.16)	10.50 (5.86)	2.17 (0.98)	32.83 (10.68)
**Overall mean (SD)**	**26.63 (6.74)**	**17.34 (6.98)**	**2.91 (1.11)**	**43.96 (12.08)**
**Intra-class correlation (95% CI)**	**0.60 (0.27-0.81)**	**0.81 (0.66-0.91)**	**0.53 (0.03-0.81)**	**0.76 (0.57-0.89)**
