# Supplementary material for: The Quality of Internet Websites for People Experiencing Psychosis: Pilot Expert Assessment
Source: JMIR Form Res. 2022 Apr 15;6(4):e28135. doi: 10.2196/28135 (PMC9055477; doi:10.2196/28135)
Supplement: Multimedia Appendix 5 [file formative_v6i4e28135_app5.docx]

**Multimedia Appendix 5. Mean (SD) total scores on the PWQC tool and subscales**

| **Website** | **Credible** | **Currency** | **Objective** | **Available Usable** | **Design Aesthetics** | **Breadth**  **Accuracy** | **Total PWQC score** |
| --- | --- | --- | --- | --- | --- | --- | --- |
| HelpGuide | 21.33 (6.71) | 6.67 (3.01) | 20.33 (4.23) | 17.50 (3.99) | 9.33 (0.82) | 103.50 (17.55) | 178.67 (23.91) |
| Patient.co.uk | 28.33 (5.65) | 6.00 (3.58) | 24.17 (4.83) | 20.00 (0.00) | 8.00 (1.79) | 89.53 (14.18) | 176.03 (22.89) |
| NHS | 25.50 (4.28) | 6.67 (1.97) | 19.17 (4.02) | 20.00 (0.00) | 8.17 (2.04) | 95.83 (19.77) | 175.33 (25.15) |
| Schizophrenia.com | 21.22 (8.45) | 6.83 (1.60) | 22.77 (5.74) | 15.17 (4.40) | 4.50 (1.87) | 102.06 (27.49) | 172.55 (46.15) |
| Early Psychosis Prevention and Intervention Centre | 17.60 (3.44) | 2.40 (0.89) | 20.48 (3.28) | 16.40 (3.58) | 6.80 (2.28) | 107.08 (17.14) | 170.76 (15.31) |
| National Institute of Mental Health | 22.22 (5.75) | 3.67 (1.97) | 20.83 (1.60) | 18.83 (2.86) | 7.50 (1.38) | 94.67 (20.06) | 167.72 (17.10) |
| Mentalhelp.net | 18.83 (5.08) | 4.83 (3.13) | 19.17 (4.67) | 16.50 (3.99) | 6.67 (2.16) | 99.00 (29.22) | 165.00 (43.68) |
| Psych Central | 23.81 (4.65) | 6.83 (1.60) | 20.33 (4.08) | 15.00 (6.00) | 5.83 (1.17) | 88.67 (29.89) | 160.47 (43.12) |
| MedicineNet | 20.67 (7.50) | 5.33 (2.07) | 20.33 (5.24) | 17.83 (2.99) | 5.50 (2.35) | 90.33 (16.88) | 160.00 (33.19) |
| Medical News Today | 25.50 (3.89) | 6.33 (1.97) | 23.17 (4.83) | 16.50 (4.18) | 7.67 (1.51) | 73.50 (8.80) | 152.67 (16.68) |
| Mayo Clinic | 24.17 (4.12) | 7.00 (2.45) | 20.00 (5.93) | 14.67 (5.42) | 5.33 (1.03) | 81.26 (7.24) | 152.42 (17.82) |
| WebMD | 23.83 (4.02) | 5.67 (2.65) | 20.00 (3.41) | 16.67 (2.73) | 7.33 (2.16) | 78.00 (17.05) | 151.50 (24.56) |
| Wikipedia | 21.83 (8.61) | 7.33 (2.07) | 19.17 (4.49) | 18.67 (2.07) | 6.83 (1.60) | 75.50 (19.84) | 149.33 (26.85) |
| Orygen Youth Health Clinical Program | 13.60 (4.34) | 3.00 (1.67) | 20.17 (3.37) | 14.17 (5.67) | 8.50 (1.52) | 86.50 (14.99) | 145.93 (21.41) |
| Headspace | 17.83 (3.66) | 5.00 (3.03) | 19.67 (5.57) | 19.00 (2.45) | 10.00 (0.0) | 70.40 (15.44) | 141.90 (16.49) |
| News-Medical.net | 24.83 (4.92) | 6.00 (3.58) | 20.67 (4.68) | 17.17 (3.49) | 6.17 (1.94) | 59.33 (9.29) | 134.17 (21.01) |
| SANE | 22.17 (3.87) | 3.33 (2.07) | 17.83 (5.60) | 18.00 (2.76) | 8.00 (1.79) | 63.83 (19.94) | 133.17 (28.14) |
| Mental Health America | 15.67 (3.61) | 2.00 (0.00) | 18.00 (4.43) | 19.50 (0.84) | 7.00 (0.89) | 70.33 (8.64) | 132.50 (13.61) |
| Healthline | 24.00 (7.27) | 4.33 (3.20) | 19.00 (6.26) | 15.50 (4.55) | 5.83 (0.98) | 62.95 (7.80) | 131.61 (22.32) |
| About Health | 16.80 (6.98) | 5.80 (1.48) | 18.20 (3.90) | 12.80 (4.82) | 6.20 (4.02) | 71.60 (22.11) | 131.40 (37.39) |
| Better Health Channel | 20.33 (8.16) | 6.33 (1.97) | 16.83 (7.08) | 17.17 (3.60) | 6.67 (2.07) | 62.17 (9.99) | 129.50 (26.81) |
| Reach Out | 17.33 (4.84) | 4.50 (1.52) | 17.17 (3.31) | 18.00 (3.09) | 8.33 (1.97) | 62.83 (18.18) | 128.17 (26.60) |
| Brain/Behaviour Research Foundation | 19.83 (5.91) | 2.67 (1.63) | 16.67 (3.08) | 16.83 (3.82) | 6.50 (1.76) | 59.17 (16.41) | 121.67 (23.05) |
| Medline Plus | 25.50 (8.29) | 6.67 (1.63) | 14.33 (4.08) | 14.17 (2.04) | 6.67 (0.82) | 46.00 (7.38) | 113.33 (9.09) |
| Farex Free Dictionary | 10.67 (3.39) | 3.33 (2.07) | 15.00 (4.29) | 13.67 (5.99) | 4.67 (0.82) | 56.55 (19.75) | 103.89 (28.75) |
| **Mean (SD)** | **20.99 (6.68)** | **5.16 (2.62)** | **19.34 (4.82)** | **16.82 (3.96)** | **6.97 (2.09)** | **77.87 (23.20)** | **147.14 (32.00)** |
| **Intra-class correlation (95% CI)** | **0.70**  **(0.46-0.86)** | **0.69**  **(0.43-0.85)** | **0.55**  **(0.18-0.78)** | **0.54**  **(0.17-0.78)** | **0.65**  **(0.37-0.83)** | **0.81**  **(0.65-0.91)** | **0.74**  **(0.52-0.87)** |
