# Supplementary material for: The Quality of Internet Websites for People Experiencing Psychosis: Pilot Expert Assessment
Source: JMIR Form Res. 2022 Apr 15;6(4):e28135. doi: 10.2196/28135 (PMC9055477; doi:10.2196/28135)
Supplement: Multimedia Appendix 6 [file formative_v6i4e28135_app6.docx]

**Multimedia Appendix 6:**

**Website categories and PWQC and DISCERN (mean ± SD) scores against site characteristics**

**of Griffiths & Christensen (2000)**

|  | **Organisational category** | | | |  | **Editorial board** | |  | |
| --- | --- | --- | --- | --- | --- | --- | --- | --- | --- |
|  | Max^a^ | Professional | Commercial | Consumer | *P* value | Yes | No | *P* value |  |
| Credibility | 35 | 21.17  (6.67) | 21.07 (6.83) | 20.44 (6.61) | .88 | 22.96 (6.57) | 18.40 (5.93) | <.001 |  |
| Currency | 10 | 4.94  (2.57) | 5.38 (2.55) | 5.23 (2.87) | .66 | 5.94 (2.34) | 4.13 (2.62) | <.001 |  |
| Objectivity | 30 | 19.02  (4.84) | 19.57 (4.79) | 19.62 (4.94) | .78 | 19.83 (5.00) | 18.69 (4.53) | .16 |  |
| Availability  Usability | 20 | 17.22  (3.80) | 15.79 (4.36) | 17.77 (3.23) | .051 | 17.21 (3.62) | 16.30 (4.35) | .16 |  |
| Design  Aesthetics | 10 | 7.51  (1.94) | 6.21 (2.08) | 7.13 (2.11) | .003 | 7.01 (1.99) | 6.91 (2.24) | .76 |  |
| Breadth  Accuracy | 145 | 77.32 (22.73) | 75.62 (22.84) | 83.05 (24.81) | .37 | 80.40 (23.57) | 74.56 (22.45) | .13 |  |
| PWQC total | 250 | 147.17 (29.01) | 143.64 (34.45) | 153.24 (33.84) | .43 | 153.35 (32.42) | 138.98 (29.76) | .006 |  |
| DISCERN Total | 75 | 43.26 (11.21) | 42.79 (12.93) | 47.57 (12.04) | .18 | 46.58 (12.53) | 40.53 (10.61) | .002 |  |

^a^Maximum score
